# Supplementary material for: Physical Characterization and Cellular Toxicity Studies of Commercial NiO Nanoparticles
Source: Nanomaterials (Basel). 2022 May 26;12(11):1822. doi: 10.3390/nano12111822 (PMC9181923; doi:10.3390/nano12111822)
Supplement: Supplementary file 1 [file nanomaterials-12-01822-s001.zip › nanomaterials-1702457-supplementary.pdf]

nanomaterials

MDPI

Supplementary Material

Physical Characterization and Cellular Toxicity Studies of Commercial NiO Nanoparticles

Filip Kunc [1], Michael Bushell [1], Xiaomei Du [2], Andre Zborowski [2], Linda J. Johnston [1] and David C. Kennedy [1,*]

[1] Metrology, National Research Council Canada, 1200 Montreal Road, Ottawa, ON K1A 0R6, Canada; filipo.kunc@gmail.com (F.K.); mikebushell@cmail.carleton.ca (M.B.); linda.johnston@nrc-cnrc.gc.ca (L.J.J.)

[2] Energy, Mining and Environment, National Research Council Canada, 1200 Montreal Road, Ottawa, ON K1A 0R6, Canada; xiaomei.du@nrc-cnrc.gc.ca (X.D.); andre.zborowski@nrc-cnrc.gc.ca (A.Z.)

* Correspondence: david.kennedy@nrc-cnrc.gc.ca

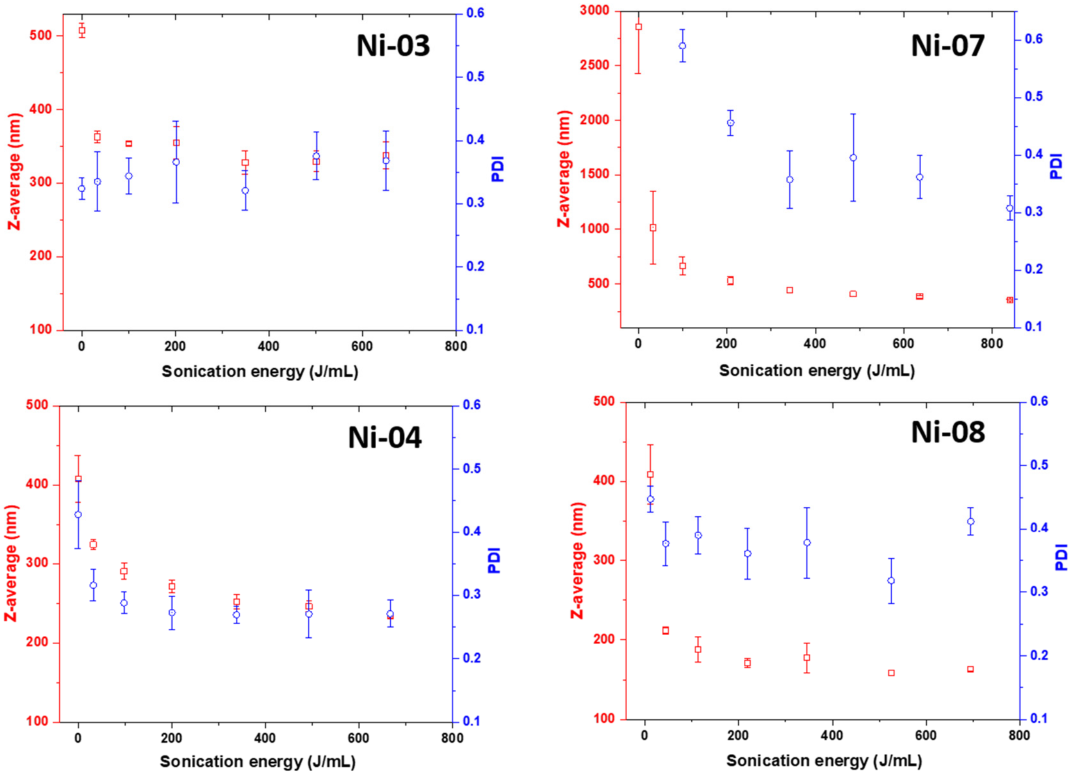

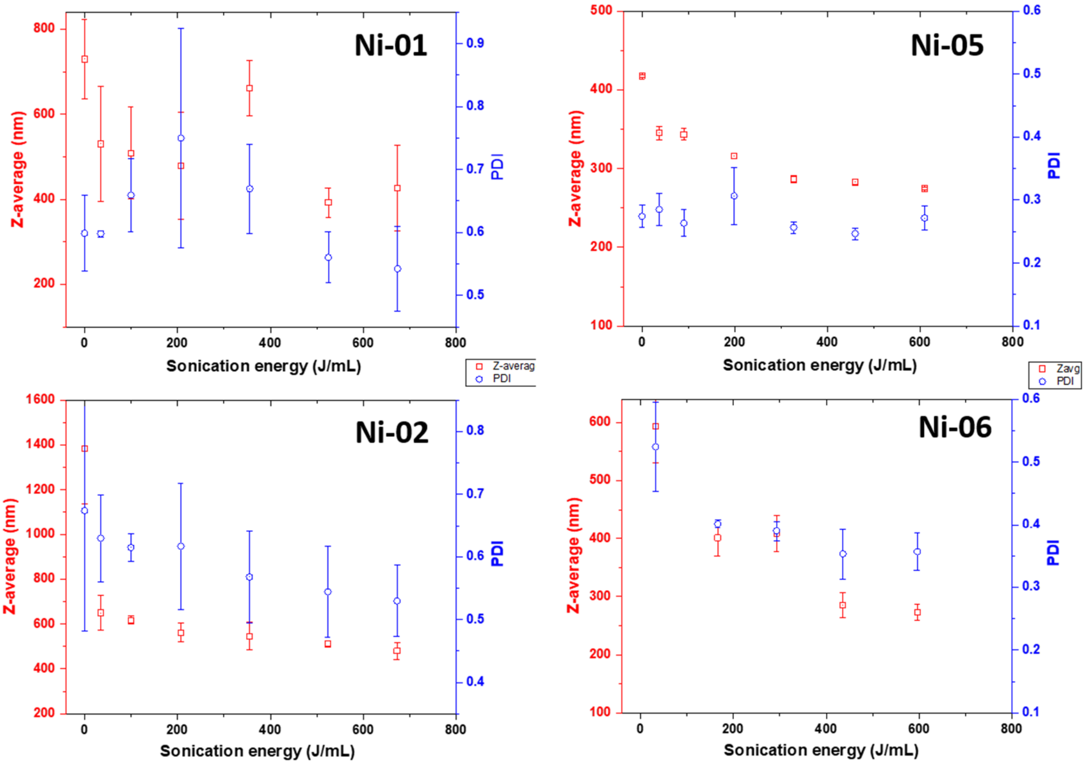

Figure S1. The evolution of Z-average (red) and polydispersity (blue) as a function of applied sonication energy in deionized water at 1 mg/mL concentration for NiO nanoparticles. The DLS measurements were done at 0.1 mg/mL.

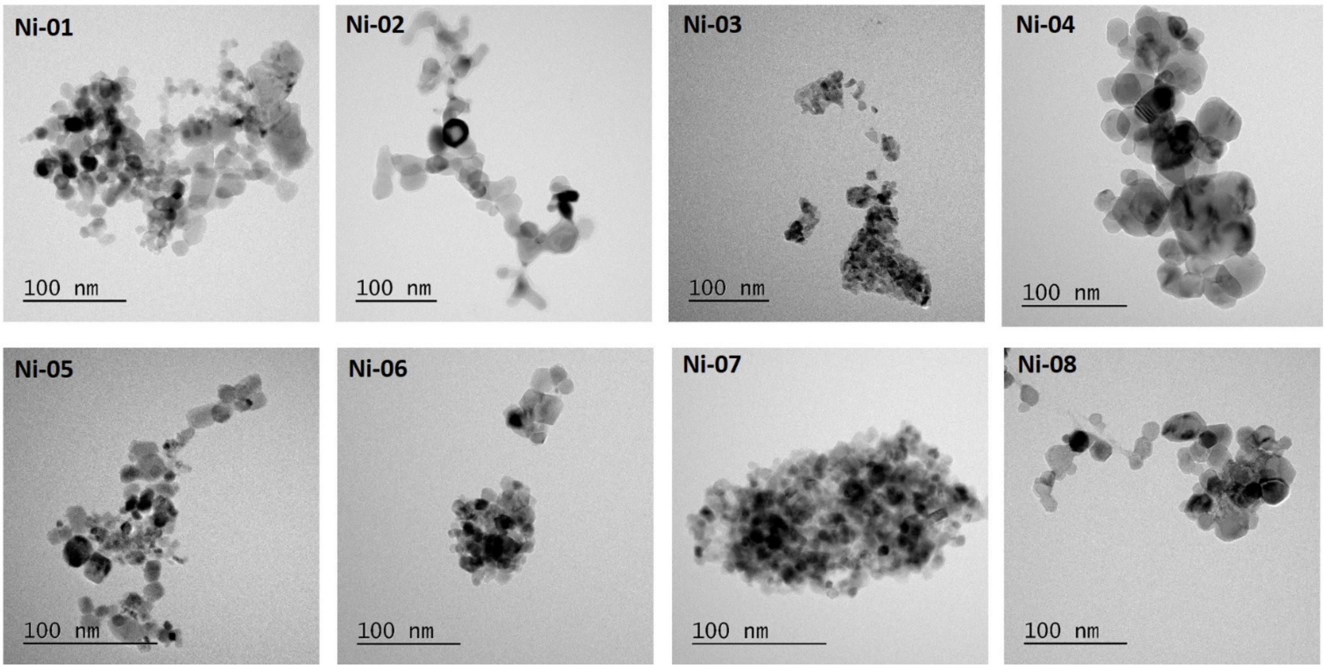

Figure S2. Representative TEM micrographs of NiO nanoparticles dispersed by sonication in water and deposited on TEM grids; images were recorded at lower magnification than those presented in Figure 1.

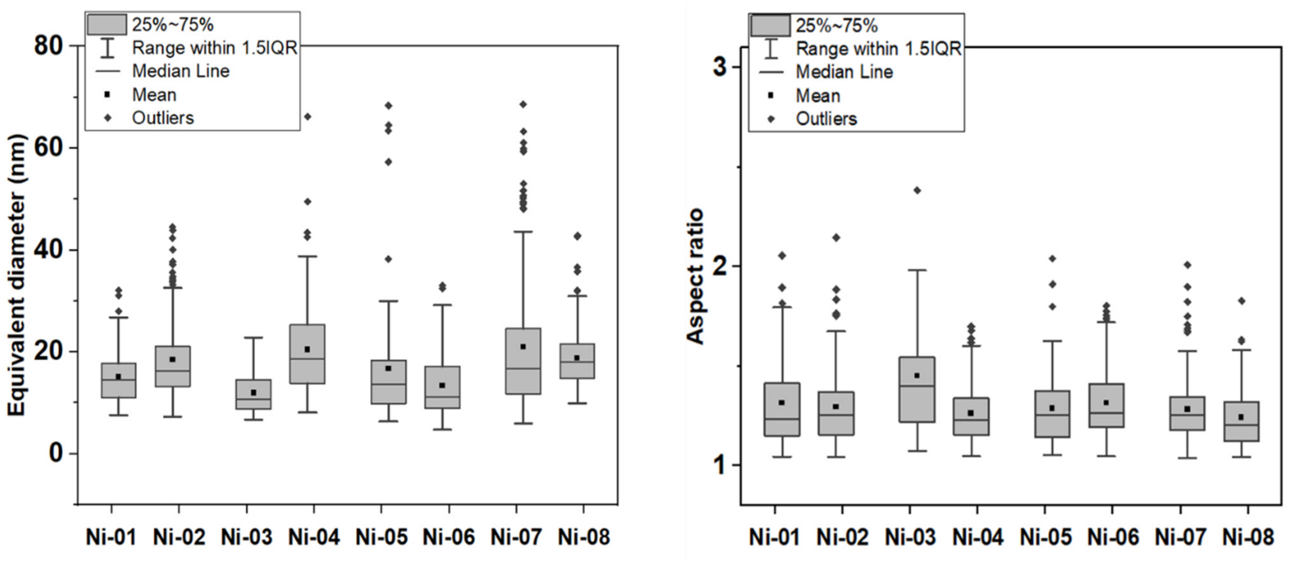

Figure S3. Box plots showing the distribution of equivalent spherical diameter and aspect ratio for the NiO nanoparticles.

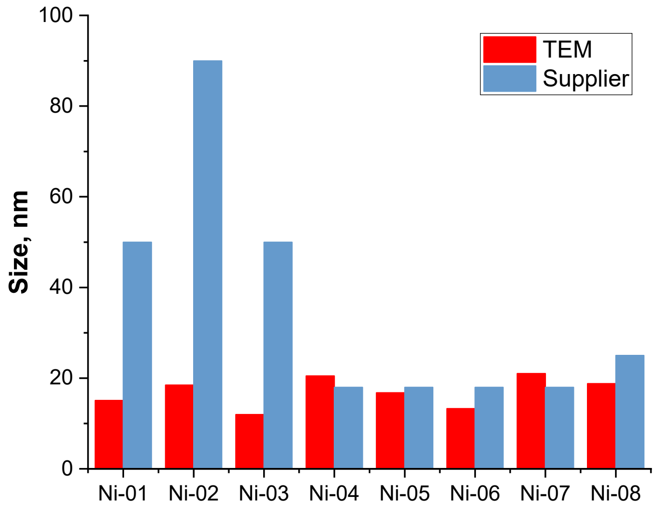

Figure S4. Comparison of particle size for NiO nanoparticles as measured by TEM (mean equivalent diameter) and reported by the manufacturer. The value reported by the manufacturer for Ni-03 is an upper limit.

Table S1. Summary of ICPMS data for NiO samples. Only metal impurities making up at least 0.1% of the total mass are included in this table. Theoretical Ni % for pure nickel oxide is 78.6%.

Ni-01	43.9	Mn (19.1%) Co (3.5%) Cr (0.7%) Bi (2.9%)
Ni-02	73.4	Mn (1.5%)
Ni-03	68.5	-
Ni-04	76.1	-
Ni-05	51.5	-
Ni-06	55.2	-
Ni-07	62.7	-

Ni-08	71.4	Co (0.3%) Ca (0.1%)

Table S2. Summary of DLS values in cell culture media over time. PDI values are reported for samples where the hydrodynamic diameter was less than 1 micron.

	**start**		**24 h**		**48 h**		**72 h**	
	**Z-average (nm)**	**PDI**	**Z-average (nm)**	**PDI**	**Z-average (nm)**	**PDI**	**Z-average (nm)**	**PDI**
Ni-O1	>1000	-	>1000	-	>1000	-	>1000	-
Ni-O2	>1000	-	>1000	-	>1000	-	>1000	-
Ni-O3	550±50	>0.4	>1000	-	>1000	-	>1000	-
Ni-O4	>1000	-	>1000	-	>1000	-	>1000	-
Ni-O5	>1000	-	>1000	-	>1000	-	>1000	-
Ni-O6	>1000	-	>1000	-	>1000	-	>1000	-
Ni-O7	750±50	>0.4	>1000	-	>1000	-	>1000	-
Ni-O8	550 ±50	>0.4	780±50)	0.85±0.05	650±60	0.76±0.02	540±50	0.90±0.03

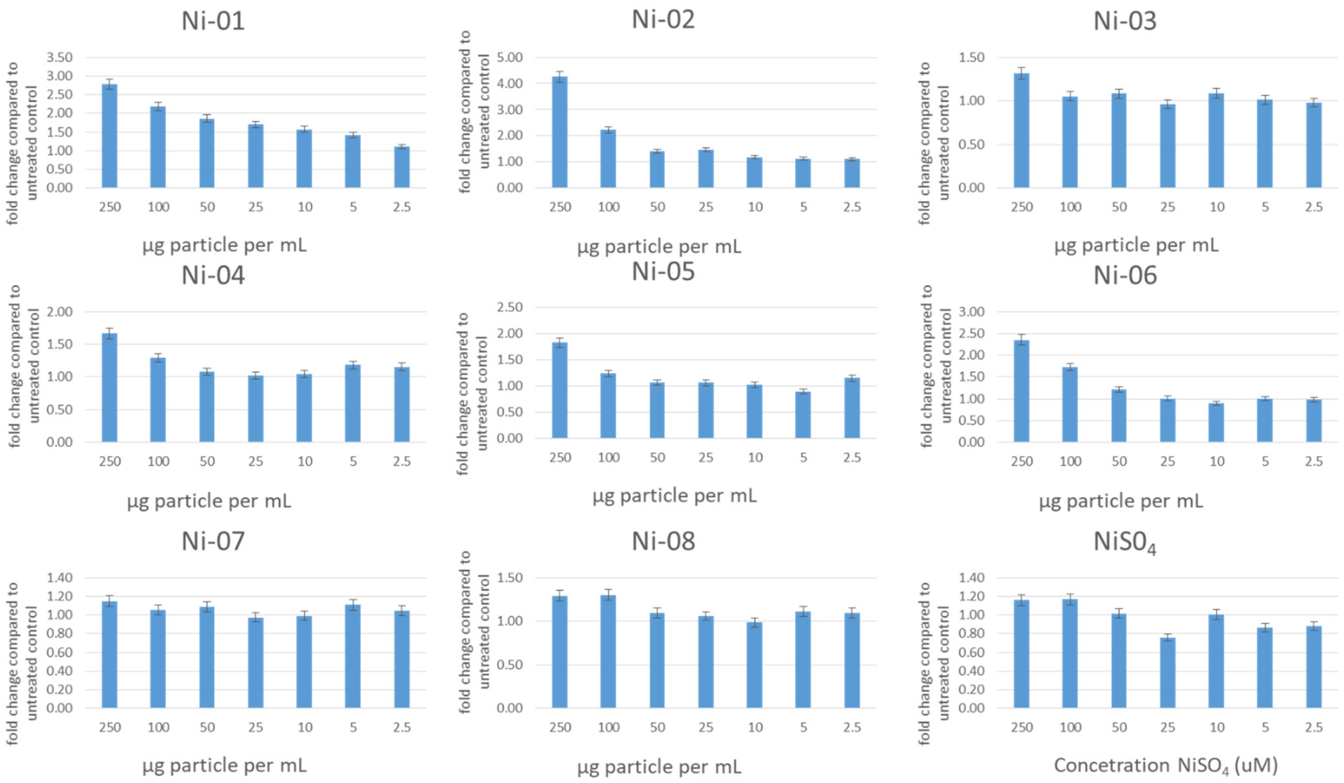

Figure S5. DCFDA values from A549 cells treated with NiO nanoparticles suspended in cell culture media spanning 2.5–250 µg/mL after 24h treatment.

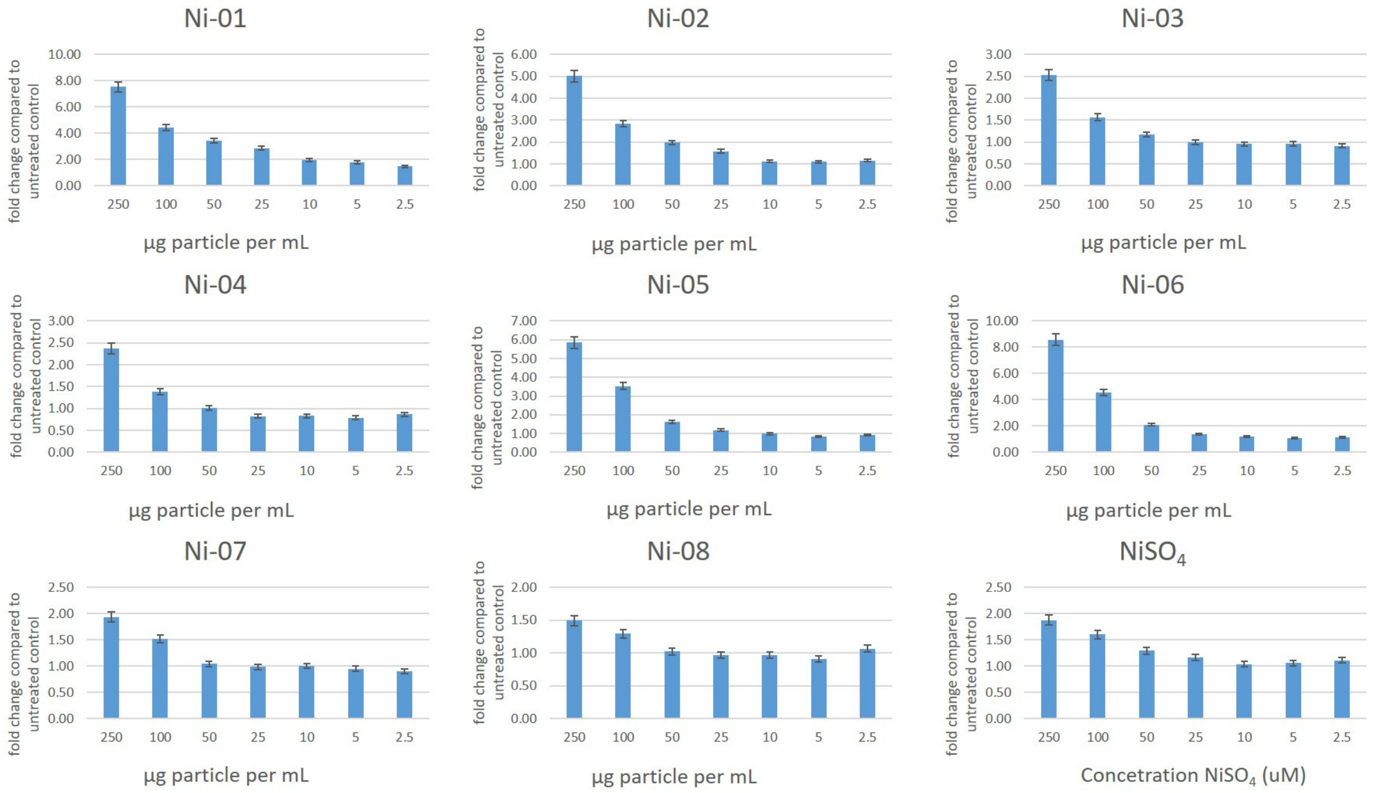

Figure S6. DCFDA values from J774A.1 cells treated with NiO nanoparticles suspended in cell culture media spanning 2.5–250 µg/mL after 24h treatment.
